# Substance Use Workforce Training Needs during Intersecting Epidemics: An Analysis of Events Offered by a Regional Training Center from 2017–2020

**DOI:** 10.21203/rs.3.rs-956280/v1

**Published:** 2021-10-11

**Authors:** Kelli Scott, Mika D.H. Salas, Denise Bayles, Raymond Sanchez, Rosemarie A Martin, Sara J Becker

**Affiliations:** Brown University; Brown University School of Public Health; Brown University School of Public Health; Brown University School of Public Health; Brown University School of Public Health; Brown University School of Public Health

**Keywords:** practice-based evidence, addiction, Technology Transfer Center, technical assistance

## Abstract

**Background:**

Intersecting opioid overdose, COVID-19, and systemic racism epidemics have brought unprecedented challenges to the addiction treatment and recovery workforce. From 2017–2020, the New England Addiction Technology Transfer Center (ATTC) collected data in real-time on the training and technical assistance (TA) requested and attended by the front-line workforce. This article synthesizes practice-based evidence on the types of TA requests, topics of TA, attendance numbers, and socio-demographics of TA attendees over a 3-year period spanning an unprecedented public health syndemic.

**Methods:**

We assessed TA events hosted by the New England ATTC using SAMHSA’s Performance Accountability and Reporting System post-event survey data from 2017–2020. Events were coded by common themes to identify the most frequently requested training types/topics and most frequently attended training events. We also evaluated change in training topics and attendee demographics over the three-year timeline.

**Results:**

A total of 258 ATTC events reaching 10,143 participants were analyzed. The number of TA events and attendance numbers surged in the 2019–2020 fiscal year as TA events shifted to fully virtual during the COVID-19 pandemic. The absolute number of opioid-related events increased, but the relative proportion remained stable over time. The relative proportions of events and attendance rates focused on evidence-based practice and health equity both increased over the 3-year period, with the largest increase after the onset of the pandemic and the murder of George Floyd. As events shifted to virtual, events were attended by providers with a broader range of educational backgrounds.

**Conclusions:**

Results of the current analysis indicate that the demand for TA increased during the pandemic, with a prioritization of TA focused on evidence-based practice and health equity. The practice-based evidence generated from the New England ATTC may help other training and TA centers to anticipate and nimbly respond to the needs of the workforce in the face of the intersecting epidemics.

## Introduction

In recent years, the United States addiction treatment and recovery workforce has faced unprecedented and intersecting public health crises: the opioid overdose epidemic, the COVID-19 pandemic, and national reckoning with systemic racism. Opioid overdose deaths rose from 47,600 in 2017 (1) to 49,860 in 2019 (2), resulting in overdose fatalities outpacing deaths due to car accidents and violent crime (3). The COVID-19 pandemic, declared a national emergency in March 2020, further exacerbated overdose risk and precipitated a spike in overdose deaths in 2020 (4). Concurrently, the United States faced national reckoning of the pervasive issue of systemic and structural racism, as COVID-19 and barriers to essential harm reduction services disproportionally affected racial/ethnic minority communities (5).

Throughout this syndemic, federally-funded purveyors of training and technical assistance (TA) have been on the front-lines helping agencies and their workforce to adopt and implement evidence-based behavioral health practices. Of existing TA providers, the Addiction Technology Transfer Centers (ATTCs), established in 1993, represent the longest running and most widely studied behavioral health training and technical assistance (TA) initiative (6). Funded by the Substance Abuse and Mental Health Services Administration (SAMHSA), the ATTC network consists of 10 regional centers, tightly coordinated by a national office, that each serve as a multidisciplinary resource for those working in the addiction treatment and recovery service fields (7).

Each regional ATTC works with expert trainers to provide three different levels of TA that vary in their objectives and duration: basic, targeted, and intensive (8). TA is defined as a tailored approach to providing implementation support to, and increasing capacity for, continuous quality improvement (9). Training is considered a discrete activity that can be included as part of any TA effort, guided by extensive evidence that strategies beyond training are required for practice implementation and organizational change (10). Basic TA focuses on information dissemination to large audiences with the goal to build awareness and/or knowledge about an evidence-based service. Targeted TA provides tailored support for specific populations or settings to foster skill development and readiness to implement specific evidence-based services. Intensive TA provides ongoing, customized consultation specific to communities, organizations, or systems aimed to support full incorporation of a new practice in real-world settings. Decisions about which TA type to provide to a specific community agency are based on circumstances, need, and appropriateness (11).

The ATTC network has not only been at the forefront of providing TA in evidence-based practice, but has also been a leader in generating practice-based evidence by synthesizing data on TA provision and engagement in real-time (12). Recent work pooling data from the ATTC network with other federally-funded TA purveyors indicated that there was an increase in the number and reach of TA events nationwide in the six-months following COVID-19 social distancing orders relative to the six-months prior (13). In addition, there was a surge in requests for basic and targeted TA, as agencies sought access to rapid information (14). These studies focused on general themes across disparate TA networks from 2019–2020, which limited detection of longer-term, addiction-specific trends.

The New England ATTC, one of the original ATTCs established in 1993, has been systematically tracking provision of and attendance at TA events since 2017. The New England ATTC serves a region (e.g., Rhode Island, Massachusetts, Connecticut, New Hampshire, Vermont, Maine) that has been at the epicenter of the opioid overdose epidemic (15,16), and at the forefront of the national response to the COVID-19 pandemic (17). As a result, the New England ATTC has generated vital practice-based evidence regarding the evolving needs of the addiction treatment and recovery support workforce in the six-state region.

This paper aims to advance knowledge on workforce training needs that emerged during the syndemic by examining the following New England ATTC metrics from 2017–2020: a) frequency of TA types delivered (basic, targeted, or intensive); b) most frequently delivered and highly attended TA topics; and c) shifts in TA requests over time. Based on national trends, we hypothesized that there would be an increase in TA requests from 2017–2020, driven by an increase in requests for basic and targeted TA. We also expected to document a rise in requests for TA on opioid-related topics and on health equity, particularly during 2019–2020 fiscal year following the murder of George Floyd (18) and during the spike in overdose deaths (4). By generating practice-based evidence, this study may help purveyors of TA to nimbly adapt and better anticipate the types of support that are the most beneficial and timely for the workforce in times of crisis.

## Methods

### Data Extraction

Event attendance and attendee socio-demographic data were extracted from SAMHSA’s Performance Accountability and Reporting System (SPARS) over three fiscal years (October 2017 to September 2020): attendance data were extracted from Event Description Forms and socio-demographic data were extracted from the Government Performance and Results Act (GPRA) post-event forms. GPRA post-event forms were administered to all event attendees, but framed as optional. TA titles and descriptions were drawn from the New England ATTC’s FileMaker® tracking system. Events in the FileMaker® system that were excluded from the final dataset included activities that were not classified as TA (i.e., meetings) or not formally organized by the New England ATTC (i.e., events coordinated by other ATTCs or TA purveyors).

### Event Coding

The topics and types of TA events were qualitatively coded using a reflexive, team-based content analysis approach (19) involving three members of the New England ATTC team. As a first step, the coding team reviewed the list of TA event titles in its entirety. The team then generated a preliminary list of event topics and an initial set of topic definitions in a coding dictionary

Two rounds of coding were completed to assign topic and type codes to the TA activities. In the first round, a primary coder (MS) independently coded all event topics. A second coder (KS) independently double coded 20% of all events. A third coder (SB) was consulted as needed throughout the coding process to add emergent topics and definitions to the coding dictionary. Once independent coding was completed, the three coders met to identify discrepant codes, to make final consensus coding determinations, and to organize codes into broader TA topics.

The final list of TA topics used for coding included: Evidence-Based Practices (EBPs), Provider Self Care, Leadership Development, Health Equity, Stigma, and Consumer Needs. Table 1 presents definitions of each topic. The coders also indicated whether each event was opioid-, justice-, or COVID-related. Following completion of the first round of coding focused on topics, a second round was conducted to classify TA type for each event (i.e. basic, targeted, or intensive TA). Coding continued until the team obtained 100% consensus.

### Data Analysis

Data extracted from SPARS and FileMaker® were integrated into a single dataset and analyzed using SPSS software (20). Descriptive statistics were run to identify the most frequent TA topic and type codes and the most frequently attended topics across the three-year period. Changes in TA topics and attendance across years were evaluated using Chi-squared analyses with Cramer’s *V* used to indicate the effect size. Consistent with well-established standards (21,22), Cramer’s *V* values were interpreted as: < .10: little or no effect,.10–.19: small effect,.20 – .29: moderate effect, and ≥ .30: strong effect.

## Results

A total of 10,695 participants attended 345 New England ATTC TA events over the three-year period, of which 258 events (75%) were retained for this analysis. Eighty-seven events with a total of 552 attendees were excluded (i.e., meetings or coordinated by other ATTCs). The final analytic sample for total attendance included 10,143 attendees (95% of participants). Demographic information was available from 6,643 attendees (66% of the analytical sample) who completed GPRA post event forms.

### Participant Demographics

Table 2 presents socio-demographic data. Of the TA attendees with available data, respondents predominantly identified as female (69.7%), and White (72.6%) with the next largest identification categories being Black (7.7%), Multi-Race (5.5%), and Hispanic (5.5%). Nearly half of participants held a Bachelor’s Degree or lower (47.3%), with the other half holding a Master’s Degree or higher (52.7%%). Attendees represented over 30 professions, with the majority identifying as behavioral health or substance use treatment providers (50.6%).

Over the three-year period, the socio-demographics of the workforce attending TA events shifted, with significant associations between grant year and gender, race/ethnicity, participant training/education background, and participant primary profession. Analyses revealed shifts in the attendee composition in terms of gender, race/ethnicity, and training background that were significant but in the trivial range (Cramer’s *V* < .10), whereas the shift in primary profession was moderate (Cramer’s *V*=.23). Attendees’ primary professions diversified across the three-year period, with greater representation among peer community support providers, education professionals, and students.

### Training Participants/Regions

Of the 258 TA events analyzed, sixty-nine percent of TA events were face-to-face and the remainder were virtual. Over the first two years, the New England ATTC provided TA both in-person and virtually. Midway through the third year (March 2020), all TA was transitioned to fully virtual due to the COVID-19 pandemic.

Table 3 presents TA event counts by state and by type. TA occurred across all six New England states, with the majority of trainings offered to the full New England region (31.4%), followed by New Hampshire (16.7%) and Connecticut (14.3%). Notably, regional coverage was fairly stable, with no significant association between fiscal year and TA by state.

### Frequency of TA Types Offered

Of the 258 events analyzed, most were categorized as targeted TA (76.4%). Intensive (18.6%) and basic TA (5.0%) were offered far less frequently. Over the three-year period, the total number of events decreased by about 10% and the TA types shifted. The proportion of events classified as targeted TA decreased by 28%, whereas the proportion classified as intensive TA increased by 23%. Chi squared analyses revealed significant differences in TA types across the fiscal years, and these changes were moderate in size (Cramer’s *V* = 0.20; see Table 3).

### Most Frequently Offered and Attended Training Topics

Across all three years, the most frequently requested TA topics and the most heavily attended included EBPs (41.5% of events, 30.9% of attendees; see Table 4), Consumer Needs (27.5% of events, 34.8% of attendees), and Health Equity (14.0% of events, 20.6% of attendees). EBPs, Health Equity, and Consumer Needs were most frequently requested as targeted TA (72.0%, 77.8%, and 81.7% were targeted TA, respectively), though EBP events were also often requested as intensive TA (27.1% intensive). Across TA categories, 21.3% of events were classified as opioid-related, 13.6% were classified as justice related, and 2.3% were classified as COVID-related.

Within the three most popular event categories, further patterns emerged. Of the EBP events, the most popular interventions were medication for opioid use disorder (29.9%) and motivational interviewing (29.0%). Within the Consumer Needs events, about two-thirds (69.0%) provided general substance use education, with topics such as diagnosing substance use disorders, recognizing co-occurring mental health disorders, and applying general clinical skills (e.g. group counseling delivery). Finally, more than half (52.8%) of the Health Equity events involved training in effectively working with specific underserved populations (e.g. sexual and gender minorities, Hispanic and Latino populations). Other popular Health Equity topics included cultural humility (33.3%) and use of the Culturally and Linguistically Appropriate Services (CLAS) standards (13.9%).

**Figure 1:**
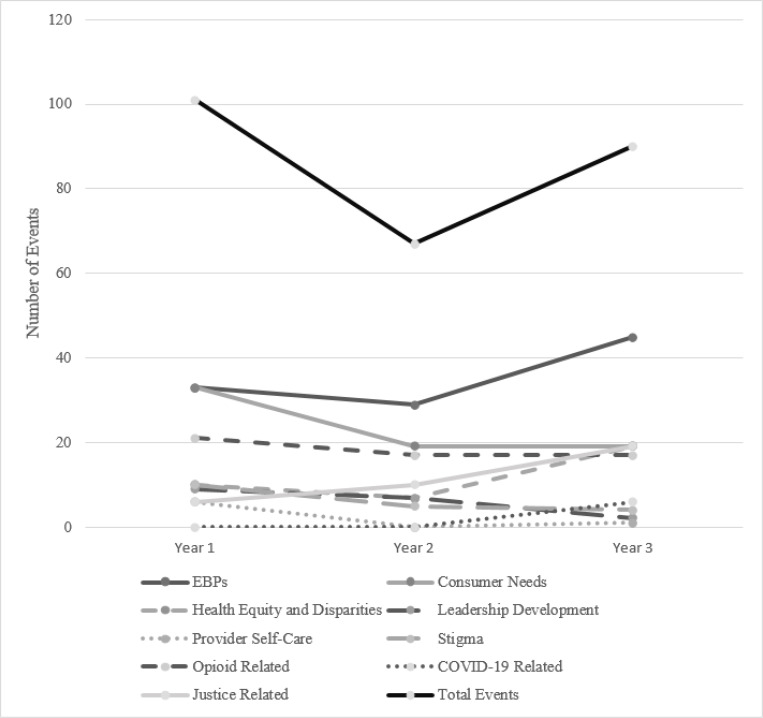
New England ATTC events by topic over a 3-year period.

**Figure 2:**
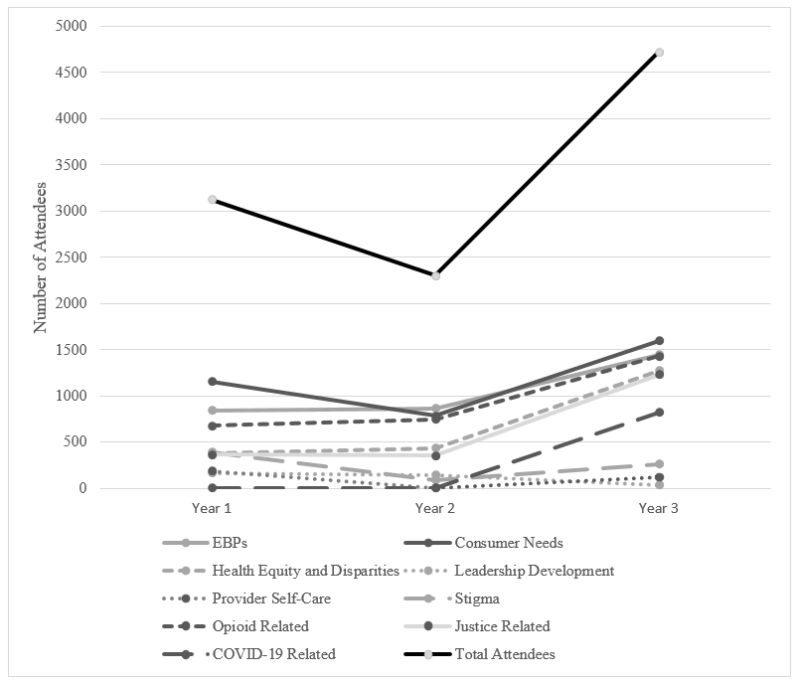
Total attendees at New England ATTC training topics across three years.

As shown in Figures 1 and 2, the total number of events declined substantially from Year 1 to Year 2 and then rebounded somewhat in Year 3 (to 90% of the Year 1 level). The number of attendees similarly declined from Year 1 to Year 2, but then jumped up substantially in Year 3 (to 150% of the Year 1 level) as events shifted to virtual delivery during the COVID-19 pandemic.

Analyses of the relative proportions of events in each year revealed moderate increases in the EBP and Health Equity categories. By contrast, there were decreases in the proportion of events in all other topics (Cramer’s *V* = 0.22). Similar trends were found in the proportion of attendees; EBP and Health Equity events had increased attendance (3.5% and 14.6% increases, respectively), while all other topics had decreased attendance (see Table 4).

## Discussion

The current study provides practice-based evidence from one of the longest operating TA purveyors in the behavioral health field during an unprecedented constellation of public health crises. Consistent with hypotheses, there was an increase in the number of TA events and attendees from the first (2017–2018) to final (2019–2020) year, with a marked jump during the pandemic as events were offered fully virtually. This increase corresponded with a broadening of event reach to providers from diverse educational backgrounds. Also congruent with hypotheses, the absolute number of events coded as opioid-related increased, although the proportion of events with this code remained relatively stable over time. The stability of opioid-related requests may have reflected the creation of the Opioid Response Network, a national network of SAMHSA-funded centers specifically focused on opioid-related technical assistance, in February 2018 (23). Finally, there was an increase (both in terms of absolute numbers and relative proportions) in the number and attendance of events focused on health equity, which corresponded with a period of heightened consciousness around systemic racism. These findings were consistent with analyses of data from multiple TA purveyors in which the overall number and reach of events surged after the announcement of social distancing orders, with health equity events having the highest attendance in the months immediately following the murder of George Floyd (14).

Counter to hypotheses, the increase in TA events was associated with an increase in intensive TA and an accompanying decrease in targeted TA events. This finding directly contradicts the results of the national study (14) of over 40 TA purveyors, which found that provision of basic and targeted TA surged after social distancing orders, and that intensive TA only accounted for 5% of all events. In the current sample, the rise in intensive TA reflected an overall increase in the provision of TA focused on evidence-based practices, with medication for opioid use disorder and motivational interviewing representing the most popular topics.

The New England ATTC’s ability to increase intensive TA offerings while other TA purveyors were providing predominantly basic or targeted support is noteworthy, especially given prior research indicating that long-term, ongoing TA is associated with better outcomes than one-shot, time-limited efforts (9). Furthermore, research on high quality TA provision has suggested that intensive TA should provide frequent, ongoing opportunities for experiential learning (24,25). The ability of the New England ATTC to provide intensive TA was likely driven by a multitude of factors including characteristics of providers in New England, characteristics of the interventions for which TA was requested (medication for opioid use disorder, motivational interviewing), and the inner setting of organizations engaging in intensive TA (26,27). The New England ATTC is also one of the only federally-funded TA purveyors with a well-established and empirically supported multi-level TA strategy: the New England ATTC’s Science to Service Laboratory combines didactic training, performance feedback, and external facilitation, and has been shown to be significantly more effective in promoting the adoption of evidence-based practice than training as usual (28–30). Having an established TA strategy likely facilitated the steady provision of intensive TA throughout the pandemic.

The practice-based evidence generated in this report is limited by the reliance on event level data, which only reveals whether TA was provided and not whether it was effective. In addition, as is true of all secondary data analysis, the quality of data analyzed is only as strong as the data entered. It is possible that some TA events were not recorded or were tracked inaccurately in the New England ATTC FileMaker system. Finally, the reliance on event titles and descriptions to discern specific TA topics might not have fully captured the focus of events.

## Conclusions

Overall, the practice-based evidence generated herein indicates that a regional federally funded TA center experienced a surge in requests for TA during the COVID-pandemic, driven by requests focused on evidence-based practice and health equity as well as requests that were intensive in nature. Findings from this analysis can help purveyors of TA to anticipate workforce development needs during the ongoing COVID-19 pandemic and future national crises. Future work should examine factors that predict TA purveyors’ ability to provide effective intensive TA.

## Supplementary Material

Supplement 1

## Figures and Tables

**Table 1:** Definitions of Technical Assistance (TA) topics.

Topic	Definition
EBPs	TA activities focused on substance use interventions supported by research, including medications for opioid use disorder, motivational interviewing, contingency management, and trauma-informed care
Provider Self-Care	TA events focused on compassion fatigue, burnout, and self-care practices for providers caring for clients with substance use disorders
Leadership Development	TA events focused on training in both clinical supervision and leadership skills
Health Equity and Disparities	TA activities focused on topics including cultural humility and providing culturally and linguistically appropriate treatment services
Stigma	TA events focused on providing education about and reducing the stigma associated with substance use disorders
Consumer Needs	TA events related to building general knowledge of substance use/substance use treatment such as the etiology and epidemiology of addiction

**Table 2:** Sociodemographic information for participants who completed post-event forms over a 3-year period (N = 6,643).

Demographic Variable	Year 1	Year 2	Year 1	Change Across Years (*X*^2^)	Cramer’s *V*
	10/2017–9/2018	10/2018–9/2019	10/2019–9/2020		
	N(%)	N(%)	N(%)		

Gender				45.9***	0.06

Male	737 (26.8%)	459 (25.0%)	449 (21.8%)		

Female	1,850 (67.3%)	1,235 (67.3%)	1,544 (75.1%)		

Transgender	4 (0.1%)	3 (0.2%)	9 (0.4%)		

None of these	0 (0%)	1 (0.1%)	11 (0.5%)		

Missing	158 (5.7%)	138 (7.5%)	44 (2.1%)		

Race				74.7***	0.08

American Indian/Alaska Native	17 (0.6%)	4 (0.2%)	15 (0.7%)		

Asian	32 (1.2%)	46 (2.5%)	46 (2.2%)		

Black	191 (6.9%)	170 (9.3%)	152 (7.4%)		

Native Hawaiian/Pacific Islander	9 (0.3%)	2 (0.1%)	8 (0.4%)		

White	2,027 (73.7%)	1,239 (67.5%)	1,555 (75.6%)		

Hispanic	127 (4.6%)	98 (5.3%)	140 (6.8%)		

Mixed Race	159 (5.8%)	137 (7.5%)	72 (3.5%)		

Missing	187 (6.8%)	140 (7.6%)	69 (3.4%)		

**Education**				94.0***	0.09

Less than high school	10 (0.4%)	5 (0.3%)	3 (0.1%)		

High school diploma, GED, Some College	341 (12.4%)	183 (10.0%)	322 (15.7%)		

Associate’s degree	196 (7.1%)	109 (5.9%)	109 (5.3%)		

Bachelor’s degree	691 (25.1%)	420 (22.9%)	617 (30.0%)	

Master’s degree	1,163 (42.3%)	795 (43.3%)	823 (40.0%)	

Doctoral Degree	186 (6.8%)	174 (9.5%)	91 (4.4%)	

Other	39 (1.4%)	33 (1.8%)	41 (2.0%)	

Missing	123 (4.5%)	117 (6.4%)	51 (2.5%)	

**Profession**			658.6***	0.23

Behavioral Health/Substance Use Treatment Provider (e.g. Counselor, Addictions Professional)	1,514 (55.1%)	933 (50.8%)	916 (44.5%)	

Medical Treatment Provider (e.g. Physician, Psychiatrist, Nurse)	299 (10.9%)	337 (18.4%)	168 (8.2%)	

Peer/Community Support Provider (e.g. Recovery Specialist, Community Health Worker)	153 (5.6%)	88 (4.8%)	187 (9.1%)	

Education (e.g. Health Educator, Researcher)	62 (2.3%)	35 (1.9%)	82 (4.0%)	

Student	0 (0%)	16 (0.9%)	245 (11.9%)	

Law Enforcement Professional (e.g. Parole Officer, Prison Staff)	97 (3.5%)	61 (3.3%)	67 (3.3%)	

Business Administrator	35 (1.3%)	28 (1.5%)	37 (1.8%)	

Other	378 (13.8%)	184 (10.0%)	261 (12.7%)	

Missing	211 (7.7%)	154 (8.4%)	94 (4.6%)	

Note.

* *p*<0.05

** *p*<0.01

*** *p*<0.001

**Table 3:** Coverage and type of Technical Assistance (TA) events over 3-year period.

Category	Year 1	Year 2	Year 3	Change Across Years (*X*^2^/% Change)	Cramer’s *V*
	10/2017–9/2018	10/2018–9/2019	10/2019–9/2020		
	N(%)	N(%)	N(%)		

**Regional Coverage**				16.2 n.s.	0.18

Regional	39 (38.6%)	14 (20.9%)	28 (31.1%)		

New Hampshire	16 (15.8%)	10 (14.9%)	17 (18.9%)		

Connecticut	13 (12.9%)	15 (22.4%)	9 (10.0%)		

Rhode Island	12 (11.9%)	9 (13.4%)	15 (16.7%)		

Massachusetts	12 (11.9%)	9 (13.4%)	8 (8.9%)		

Maine	7 (6.9%)	6 (9.0%)	11 (12.2%)		

Vermont	2 (2.0%)	4 (6.0%)	2 (2.2%)		

**Type of TA**				21.0***	0.20

Basic TA	3 (3.0%)	3 (4.5%)	7 (7.8%)	+4.8%	

Targeted TA	91 (90.1%)	50 (74.6%)	56 (62.2%)	−27.9%	

Intensive TA	7 (6.9%)	14 (20.9%)	27 (30.0%)	+23.1%	

Note. n.s. = not significant

*** p < .001

**Table 4:** Frequency of and attendance at Technical Assistance (TA) events by topic.

Topic	Year 1	Year 2	Year 3	Change Across Years (*X*^2^/% Change)	Cramer’s *V*
	10/2017–9/2018	10/2018–9/2019	10/2019–9/2020		
	N(%)	N(%)	N(%)		
	# Attendees	# Attendees	# Attendees		

**Frequency of Events**				24.0**	0.22

EBP	33 (32.7%)	29 (43.3%)	45 (50.0%)	+17.3%	

Consumer Needs	33 (32.7%)	19 (28.4%)	19 (21.1%)	−11.6%	

Health Equity and Disparities	10 (9.9%)	7 (10.4%)	19 (21.1%)	+11.2%	

Leadership Development	9 (8.9%)	7 (10.4%)	2 (2.2%)	−6.7%	

Provider Self Care	6 (5.9%)	0 (0%)	1 (1.1%)	−4.8%	

Stigma	10 (9.9%)	5 (7.5%)	4 (4.4%)	−5.5%	

**Attendance at Events**					

EBP	841 (27.0%)	857 (37.2%)	1440 (30.5%)	+3.5%	

Consumer Needs	1156 (37.1%)	782 (33.9%)	1596 (33.8%)	−3.3%	

Health Equity and Disparities	383 (12.3%)	435 (18.9%)	1268 (26.9%)	+14.6%	

Leadership Development	164 (5.3%)	145 (6.3%)	32 (0.7%)	−4.6%	

Provider Self Care	189 (6.1%)	0 (0%)	121 (2.6%)	−3.5%	

Stigma	386 (12.4%)	85 (3.7%)	263 (5.6%)	−6.8%	

Note.

** = p<0.01

## Abbreviations

ATTC: Addiction Technology Transfer Center
TA: Technical assistance
